# Supplementary material for: Synergistic Effects of Earthworms and Plants on Chromium Removal from Acidic and Alkaline Soils: Biological Responses and Implications
Source: Biology (Basel). 2023 Jun 8;12(6):831. doi: 10.3390/biology12060831 (PMC10295528; doi:10.3390/biology12060831)
Supplement: Supplementary file 1 [file biology-12-00831-s001.zip › Supplementary Materials.pdf]

biology

MDPI

*Article*

# Synergistic Effects of Earthworms and Plants on Chromium Removal from Acidic and Alkaline Soils: Biological Responses and Implications

**Ping Liu, Yan Song, Jie Wei, Wei Mao, Jing Ju, Shengyang Zheng and Haitao Zhao**

**Table S1.** Biomass and Cr concentrations of earthworms.

**1.** Biomass and Cr concentrations of earthworms in acidic soil.

| Treatments | Earthworm Biomass | Earthworm Cr Concentration |
|---|---|---|
| LEP0 | 12.04±0.21a | 0.85±0.01c |
| LER | 12.13±0.26a | 0.82±0.01c |
| LEC | 12.19±0.11a | 0.81±0.01c |
| LPP0 | 11.99±0.11a | 0.76±0.01d |
| LPR | 12.12±0.05a | 0.76±0.01d |
| LPC | 11.97±0.09ab | 0.76±0.01d |
| HEP0 | 11.63±0.11c | 2.54±0.03a |
| HER | 11.61±0.05c | 2.52±0.01a |
| HEC | 11.69±0.06c | 2.53±0.03a |
| HPP0 | 11.72±0.05bc | 2.25±0.02b |
| HPR | 11.70±0.10c | 2.25±0.03b |
| HPC | 11.70±0.07c | 2.26±0.04b |

**2.** Biomass and Cr concentrations of earthworms in alkaline soil.

| Treatments | Earthworm Biomass | Earthworm Cr Concentration |
|---|---|---|
| LEP0 | 11.06±0.12ab | 0.72±0.01cd |
| LER | 11.10±0.15a | 0.73±0.01c |
| LEC | 11.13±0.14a | 0.70±0.01cd |
| LPP0 | 10.99±0.09ab | 0.68±0.01d |
| LPR | 11.09±0.08ab | 0.68±0.01d |
| LPC | 11.11±0.03a | 0.68±0.01d |
| HEP0 | 10.81±0.04cd | 2.28±0.03a |
| HER | 10.92±0.06bc | 2.26±0.03a |
| HEC | 10.72±0.04d | 2.26±0.03a |
| HPP0 | 10.73±0.01d | 2.06±0.02b |
| HPR | 10.71±0.02d | 2.06±0.04b |
| HPC | 10.72±0.03d | 2.07±0.02b |

**Table S2.** Biomass and Cr concentrations of Plants.

**1.** Biomass and Cr concentrations of Plants in acidic soil.

| Treatments | Plant Biomass | Plant Cr Concentration |
|---|---|---|
| LEP0 | 10.08±0.02f | 0.42±0.01d |
| LER | 8.51±0.03h | 0.10±0.01f |
| LEC | 15.47±0.09a | 0.54±0.01c |
| LPP0 | 11.22±0.08d | 0.12±0.01f |
| LPR | 13.11±0.10c | 0.49±0.01c |
| LPC | 10.04±0.05f | 0.12±0.01f |
| HEP0 | 9.46±0.63g | 1.29±0.06b |
| HER | 8.35±0.06h | 0.29±0.03e |
| HEC | 14.70±0.17b | 1.58±0.04a |
| HPP0 | 10.66±0.12e | 0.41±0.02d |
| HPR | 13.09±0.10c | 1.62±0.03a |
| HPC | 9.91±0.05f | 0.33±0.02e |

**2.** Biomass and Cr concentrations of Plants in alkaline soil.

| Treatments | Plant Biomass | Plant Cr Concentration |
|---|---|---|
| LEP0 | 13.55±0.05e | 0.41±0.01e |
| LER | 0.23±0.01gh | 0.11±0.00h |
| LEC | 15.62±0.08a | 0.48±0.01d |
| LPP0 | 0.30±0.01g | 0.12±0.01h |
| LPR | 14.97±0.11b | 0.45±0.02d |
| LPC | 0.25±0.01gh | 0.10±0.00h |
| HEP0 | 13.20±0.09f | 1.35±0.03c |
| HER | 0.13±0.01h | 0.12±0.01h |
| HEC | 14.38±0.09c | 1.64±0.03a |
| HPP0 | 0.18±0.01gh | 0.26±0.02f |
| HPR | 13.84±0.09d | 1.58±0.03b |
| HPC | 0.17±0.02h | 0.18±0.01g |

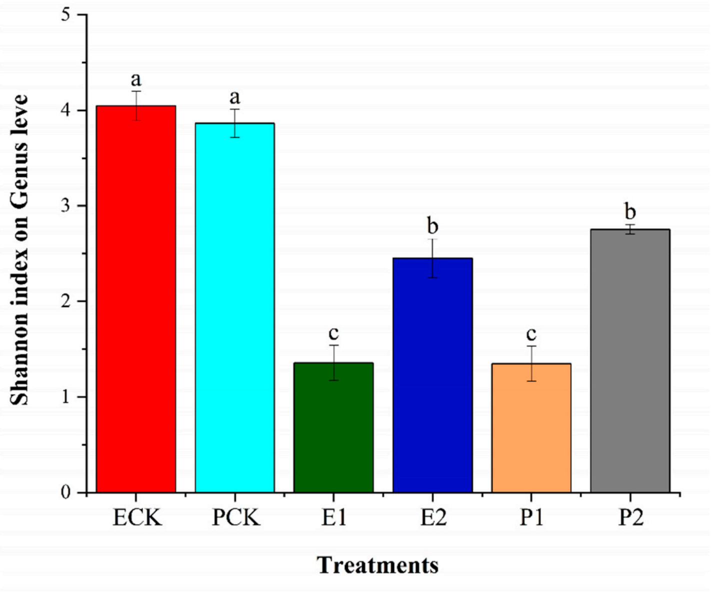


**Figure S1.** Shannon indices of genus level bacterial communities of two earthworms in acidic and alkaline soils.
